# Urinary polyaromatic hydrocarbons are associated with adult celiac disease and kidney stones: USA NHANES, 2011–2012

**DOI:** 10.1007/s11356-015-5980-7

**Published:** 2016-01-05

**Authors:** Ivy Shiue

**Affiliations:** Health and Life Sciences, Northumbria University, Northumbria University, NE1 8ST Newcastle upon Tyne, England UK; Owens Institute for Behavioral Research, University of Georgia, Athens, GA USA

**Keywords:** Polyaromatic hydrocarbons, Risk factor, Kidney, Celiac disease, Environmental health, Risk assessment

## Abstract

Links between environmental chemicals and human health have emerged over the last few decades, but the effects from polyaromatic hydrocarbons (PAH) were less studied, compared to other commonly known environmental chemicals such as heavy metals, phthalates, arsenic, phenols, and pesticides. Therefore, it was aimed to study the relationships of urinary PAH and adult digestive conditions using a large human sample in a national and population-based study in recent years. Data was retrieved from the US National Health and Nutrition Examination Surveys, 2011–2012 including demographics, self-reported health conditions, and urinary PAH. Statistical analyses included chi-square test, *t* test, survey-weighted logistic regression modeling, and population attributable risk (PAR) estimation. Of 5560 American adults aged 20–80 and included in the statistical analysis, urinary 4-hydroxyphenanthrene was significantly associated with celiac disease (odds ratio (OR) 1.61, 95 % confidence interval (CI) 1.14–2.26, *P* = 0.009). In addition, urinary 2-hydroxyfluorene (OR 1.35, 95 % CI 1.02–1.78, *P* = 0.038), 3-hydroxyfluorene (OR 1.35, 95 % CI 1.07–1.70, *P* = 0.015), 1-hydroxyphenanthrene (OR 1.48, 95 % CI 1.08–2.03, *P* = 0.017), 1-hydroxypyrene (OR 1.36, 95 % CI 1.05–1.77, *P* = 0.023), and 2-hydroxynapthalene (OR 1.25, 95 % CI 1.00–1.58, *P* = 0.054) were significantly associated with kidney stones, although not necessarily failing kidney. There were no statistically significant associations observed in the relationship of urinary PAH and liver problems, although higher levels of PAHs were observed. Urinary PAHs are associated with adult digestive conditions, although the causality cannot be established. From the research perspective, longitudinal monitoring from observational studies and experimental research understanding mechanism would be suggested. Regulation of minimizing PAHs exposure might need to be considered in future health and environmental policies.

## Introduction

### Evidence before this study

Links between environmental chemicals and human health including self-rated health, hypertension, cardiovascular disease, food allergy, oral health, emotional support, and cognitive function in American adults have emerged (Shiue 2013a, b, c, 2014, 2015a, b, c, d, e), but the effects from polyaromatic hydrocarbons (PAHs) were less studied, compared to other commonly known environmental chemicals such as heavy metals, arsenic, phenols, and phthalates. PAHs constitute a group of chemicals that people could be exposed via vehicle exhausts, asphalt, coal tar, wild fires, agricultural burning, soil, charbroiled foods, and tobacco smoke. Approximately, everyone could be exposed to PAHs on a daily basis from multiple sources. PAH pollution may have significant health implications, and the extent of damage to organisms from PAH exposure could be dependent on several factors including degrees and types of PAH exposure (Ball and Truskewycz 2013).

### Knowledge gap

Previously, animal models under a laboratory condition using rodents showed that exposure to PAHs adversely affected immunologic health (Luebke et al. 1997). However, research in this topic from a human sample has not been well conducted. Providing evidence using a human sample might help environmental health promotion in the next few years. Recently, associations of PAHs and cardiovascular, oral, emotional, and self-rated health have been observed (Shiue 2015a, b, c, d, e), but those on digestive health have not been documented. Following this context, therefore, the present study aimed to examine the relationships of urinary PAHs and adult digestive conditions using a large human sample in a national and population-based setting in recent years.

## Methods

### Study sample

As described elsewhere (Centers for Disease Control and Prevention 2012), US National Health and Nutrition Examination Surveys (NHANES) has been a national, population-based, multi-year, cross-sectional study since the 1980s. Study samples are a representative sample of the civilian, non-institutionalized US population. Information on demographics (more details via http://wwwn.cdc.gov/nchs/nhanes/2011-2012/DEMO_G.htm), serum cotinine (more details via http://wwwn.cdc.gov/nchs/nhanes/2011-2012/COTNAL_G.htm), and self-reported health conditions (more details via http://wwwn.cdc.gov/nchs/nhanes/2011-2012/MCQ_G.htm) was obtained by household interview using questionnaires. In the current analysis, the most recent study cohort in 2011–2012 with data on urinary PAHs was selected. Informed consents were obtained from participating subjects by the NHANES researchers.

### Biomonitoring

Urines were only collected in a subsample, being one third of the whole study cohort with representation (more details via http://www.cdc.gov/nchs/data/nhanes/nhanes_09_10/homeurine.pdf), to measure environmental chemical concentrations in urines among people aged 6 and above (more details via http://www.cdc.gov/nchs/nhanes/nhanes2011-2012/labdoc_g.htm). Urine specimens from urinary PAH were processed, stored under appropriate frozen (−20 °C) conditions, and shipped to the Division of Environmental Health Laboratory Sciences, National Center for Environmental Health, Centers for Disease Control and Prevention for analysis. According to the NHANES website (more details via http://www.cdc.gov/nchs/data/nhanes/nhanes_11_12/PAH_G_met.pdf), the procedure involved enzymatic hydrolysis of glucuronidated/sulfated OH-polyaromatic hydrocarbons metabolites in urine, extraction, derivatization, and analysis using isotope dilution capillary gas chromatography tandem mass spectrometry (GC-MS/MS). Ion transitions specific to each analyte and carbon-13-labeled internal standards are monitored, and the abundances of each ion are measured. Since urinary PAHs were highly skewed, they were all log transformed when performing the statistical analyses.

### Statistical analysis

Adults aged 20 and above were included in the current statistical analysis since chronic diseases were commonly reported in adults. Associations of urinary PAHs and adult self-reported digestive conditions were examined by using *t* test and survey-weighted logistic regression model, presenting with mean values, odds ratios (OR), 95 % confidence intervals (CI), and *P* values. Covariates including urinary creatinine, age, sex, ratio of family income to poverty (proxy of socioeconomic status, calculated by dividing family (or individual) income by the poverty guidelines specific to the survey year; more details via http://wwwn.cdc.gov/Nchs/Nhanes/2011-2012/DEMO_G.htm), body mass index, education level, serum cotinine (biomarker of smoking status), alcohol status (>12 drinks currently or not), and physical activity level (moderate recreational activity or not) were adjusted. In addition, population attributable risks from urinary PAHs, which significant associations were found, were calculated based on the formula introduced by Fleiss (1979). Statistical software STATA version 13.0 (STATA, College Station, Texas, USA) was used to perform all the analyses.

### Ethics consideration

Since there are only secondary data analyses employed without any participant personal information identified by extracting statistical data from the NHANES website in the present study, no further ethics approval for conducting the present study is required (more details via http://www.ethicsguidebook.ac.uk/Secondary-analysis-106).

## Results

The characteristics of 5560 American adults aged 20–80 and included in the statistical analysis are shown in Table 1. The presence of different digestive conditions in the American adult population varied since some are common while some are rare. They are shown in Table 2. In Tables 3, 4, 5, 6, 7, 8, 9, 10, and 11, associations of 10 urinary PAHs and adult digestive conditions are presented separately. Specifically, urinary 4-hydroxyphenanthrene was significantly associated with celiac disease (OR 1.61, 95 % CI 1.14–2.26, *P* = 0.009). In addition, urinary 2-hydroxyfluorene (OR 1.35, 95 % CI 1.02–1.78, *P* = 0.038), 3-hydroxyfluorene (OR 1.35, 95 % CI 1.07–1.70, *P* = 0.015), 1-hydroxyphenanthrene (OR 1.48, 95 % CI 1.08–2.03, *P* = 0.017), 1-hydroxypyrene (OR 1.36, 95 % CI 1.05–1.77, *P* = 0.023), and 2-hydroxynapthalene (OR 1.25, 95 % CI 1.00–1.58, *P* = 0.054) were significantly associated with kidney stones, although not necessarily failing kidney. However, there were no statistically significant associations observed in the relationship of urinary PAHs and liver problem, although there were trends toward higher levels of urinary PAHs in people with liver problem. In a subsequent analysis where infection and nutrients were also adjusted, the significant associations have remained (data not shown).

**Table 1:** Characteristics of the included participants aged 20–80 (*n* = 5560)

	Number (%) or mean ± SD
Age	48.9 ± 17.9
20–39	1957 (35.2)
40–59	1812 (32.6)
60–80	1791 (32.2)
Sex	
Male	2740 (49.3)
Female	2820 (50.7)
Body mass index	28.8 ± 6.9
<18.5	1103 (1.9)
18.5–24.9	1577 (28.4)
25–29.9	1684 (30.3)
30+	2196 (39.5)
Ratio of family income to poverty	
0–4.9	4199 (75.5)
5+	1361 (24.5)
Education level	
Less than 9th grade	550 (9.9)
9–11th grades	782 (14.1)
High school graduate or equivalent	1169 (21.0)
Some college or AA degree	1657 (29.8)
College graduate or above	1397 (25.2)
Serum cotinine (ng/mL)	52.1 ± 120.2
Alcohol status	
>12 drinks	3413 (72.8)
Less than 12 drinks	1275 (27.2)
Physical activity level	
Engaging moderately	2297 (41.3)
None	3262 (58.7)
Celiac disease	18 (0.3)
Liver problem	219 (3.9)
Weak/failing kidney	200 (3.6)
Kidney stones	458 (8.3)

**Table 2:** Associations between 2-hydroxyfluorene (ng/L) and adult health (*n* = 1670)

	Present	Absent	*P* value	OR (95 % CI)^a^	*P* value	Population attributable risk
Celiac disease	1073.3 (1478.6)	599.0 (1059.9)	0.208	1.48 (0.28–7.88)	0.630	–
Liver problem	705.4 (1021.3)	597.3 (1063.8)	0.414	0.93 (0.65–1.34)	0.678	–
Weak/failing kidney	567.1 (701.6)	602.5 (1072.8)	0.805	1.14 (0.81–1.61)	0.432	–
Kidney stones	641.4 (984.7)	597.5 (1070.3)	0.626	1.35 (1.02–1.78)	0.038	2.8 % (0.2–6.1 %)

^a^Adjusted for urine creatinine, age, sex, body mass index, ratio of family income to poverty, education level, serum cotinine, alcohol habit, physical activity level, and subsampling weighting

**Table 3:** Associations between 3-hydroxyfluorene (ng/L) and adult health (*n* = 1670)

	Present	Absent	*P* value	OR (95 % CI)^a^	*P* value	Population attributable risk
Celiac disease	543.9 (790.2)	294.4 (613.2)	0.252	2.03 (0.31–13.12)	0.436	–
Liver problem	339.3 (615.1)	293.9 (614.2)	0.553	1.00 (0.66–1.52)	0.994	–
Weak/failing kidney	245.7 (402.1)	297.5 (620.4)	0.535	0.98 (0.75–1.29)	0.899	–
Kidney stones	303.7 (547.2)	295.1 (621.0)	0.870	1.35 (1.07–1.70)	0.015	2.8 % (0.4–3.6 %)

^a^Adjusted for urine creatinine, age, sex, body mass index, ratio of family income to poverty, education level, serum cotinine, alcohol habit, physical activity level, and subsampling weighting

**Table 4:** Associations between 9-hydroxyfluorene (ng/L) and adult health (*n* = 1670)

	Present	Absent	*P* value	OR (95 % CI)^a^	*P* value	Population attributable risk
Celiac disease	583.8 (565.8)	518.4 (869.6)	0.832	1.40 (0.88–2.22)	0.148	–
Liver problem	590.2 (774.0)	516.3 (872.0)	0.495	0.91 (0.51–1.63)	0.736	–
Weak/failing kidney	604.8 (942.0)	515.9 (866.1)	0.448	1.38 (0.69–2.75)	0.335	–
Kidney stones	561.0 (743.9)	514.7 (880.3)	0.530	1.21 (0.98–1.50)	0.075	–

^a^Adjusted for urine creatinine, age, sex, body mass index, ratio of family income to poverty, education level, serum cotinine, alcohol habit, physical activity level, and subsampling weighting

**Table 5:** Associations between 1-hydroxyphenanthrene (ng/L) and adult health (*n* = 1670)

	Present	Absent	*P* value	OR (95 % CI)^a^	*P* value	Population attributable risk
Celiac disease	240.4 (184.3)	201.5 (286.0)	0.701	2.02 (0.71–5.71)	0.173	–
Liver problem	188.9 (172.3)	202.4 (289.3)	0.704	0.78 (0.42–1.45)	0.401	–
Weak/failing kidney	188.3 (260.1)	202.2 (286.6)	0.717	1.30 (0.72–2.34)	0.367	–
Kidney stones	217.5 (226.0)	200.2 (291.0)	0.475	1.48 (1.08–2.03)	0.017	2.5 % (0.4–5.3 %)

^a^Adjusted for urine creatinine, age, sex, body mass index, ratio of family income to poverty, education level, serum cotinine, alcohol habit, physical activity level, and subsampling weighting

**Table 6:** Associations between 2-hydroxyphenanthrene (ng/L) and adult health (*n* = 1670)

	Present	Absent	*P* value	OR (95 % CI)^a^	*P* value	Population attributable risk
Celiac disease	162.4 (153.9)	108.4 (152.2)	0.317	2.06 (0.80–5.26)	0.124	–
Liver problem	119.6 (115.4)	108.3 (153.5)	0.555	0.87 (0.46–1.65)	0.645	–
Weak/failing kidney	81.1 (83.8)	109.6 (153.9)	0.164	0.95 (0.51–1.77)	0.873	–
Kidney stones	116.5 (133.5)	107.8 (153.9)	0.500	1.43 (0.97–2.09)	0.068	–

^a^Adjusted for urine creatinine, age, sex, body mass index, ratio of family income to poverty, education level, serum cotinine, alcohol habit, physical activity level, and subsampling weighting

**Table 7:** Associations between 3-hydroxyphenanthrene (ng/L) and adult health (*n* = 1670)

	Present	Absent	*P* value	OR (95 % CI)^a^	*P* value	Population attributable risk
Celiac disease	183.5 (187.2)	122.3 (228.7)	0.450	1.82 (0.54–6.14)	0.312	–
Liver problem	134.4 (185.5)	122.1 (230.1)	0.666	0.87 (0.44–1.71)	0.666	–
Weak/failing kidney	92.3 (109.8)	123.5 (231.4)	0.314	1.00 (0.69–1.45)	0.991	–
Kidney stones	117.2 (132.0)	123.0 (236.0)	0.764	1.35 (0.99–1.85)	0.058	–

^a^Adjusted for urine creatinine, age, sex, body mass index, ratio of family income to poverty, education level, serum cotinine, alcohol habit, physical activity level, and subsampling weighting

**Table 8:** Associations between 1-hydroxypyrene (ng/L) and adult health (*n* = 1670)

	Present	Absent	*P* value	OR (95 % CI)^a^	*P* value	Population attributable risk
Celiac disease	193.1 (141.6)	202.9 (330.0)	0.933	1.30 (0.29–5.83)	0.721	–
Liver problem	233.4 (359.1)	201.8 (328.2)	0.442	1.00 (0.62–1.63)	0.988	–
Weak/failing kidney	136.9 (192.3)	205.3 (333.0)	0.127	0.80 (0.63–1.01)	0.057	–
Kidney stones	204.2 (278.9)	202.9 (334.3)	0.965	1.36 (1.05–1.77)	0.023	1.9 % (0.3–4.0 %)

^a^Adjusted for urine creatinine, age, sex, body mass index, ratio of family income to poverty, education level, serum cotinine, alcohol habit, physical activity level, and subsampling weighting

**Table 9:** Associations between 1-hydroxynapthalene (1-naphthol) (ng/L) and adult health (*n* = 1670)

	Present	Absent	*P* value	OR (95 % CI)^a^	*P* value	Population attributable risk
Celiac disease	6372.8 (7289.4)	37,428.0 (602,343.9)	0.884	1.34 (0.97–1.86)	0.076	–
Liver problem	187,972.7 (1,462,008.0)	31,118.9 (537,358.8)	0.036	0.99 (0.80–1.22)	0.930	–
Weak/failing kidney	217,324.0 (1,585,249.0)	31,089.0 (536,058.9)	0.022	0.91 (0.70–1.17)	0.436	–
Kidney stones	99,028.9 (1,035,260.0)	31,252.8 (540,238.8)	0.184	1.04 (0.88–1.24)	0.601	–

^a^Adjusted for urine creatinine, age, sex, body mass index, ratio of family income to poverty, education level, serum cotinine, alcohol habit, physical activity level, and subsampling weighting

**Table 10:** Associations between 2-hydroxynapthalene (2-naphthol) (ng/L) and adult health (*n* = 1670)

	Present	Absent	*P* value	OR (95 % CI)^a^	*P* value	Population attributable risk
Celiac disease	10,106.8 (10,404.5)	8905.0 (12,106.0)	0.779	1.49 (0.78–2.86)	0.214	–
Liver problem	11,097.9 (14,134.9)	8820.5 (12,002.4)	0.131	1.04 (0.66–1.62)	0.868	–
Weak/failing kidney	8867.4 (10,518.2)	8912.5 (12,154.7)	0.978	0.78 (0.58–1.06)	0.105	–
Kidney stones	9490.2 (10,811.0)	8839.1 (12,215.2)	0.525	1.25 (1.00–1.58)	0.054	1.3 % (0–3.0 %)

^a^Adjusted for urine creatinine, age, sex, body mass index, ratio of family income to poverty, education level, serum cotinine, alcohol habit, physical activity level, and subsampling weighting

**Table 11:** Associations between 4-hydroxyphenanthrene (ng/L) and adult health (*n* = 1670)

	Present	Absent	*P* value	OR (95 % CI)^a^	*P* value	Population attributable risk
Celiac disease	41.4 (45.2)	35.0 (51.4)	0.742	1.61 (1.14–2.26)	0.009	0.2 % (n/a)
Liver problem	48.0 (117.0)	34.5 (46.8)	0.036	0.71 (0.37–1.37)	0.291	–
Weak/failing kidney	29.0 (32.5)	35.3 (51.9)	0.361	1.14 (0.62–2.07)	0.659	–
Kidney stones	35.0 (40.5)	35.1 (52.4)	0.983	1.33 (0.95–1.86)	0.093	–

^a^Adjusted for urine creatinine, age, sex, body mass index, ratio of family income to poverty, education level, serum cotinine, alcohol habit, physical activity level, and subsampling weighting

## Discussion

### Previous research synthesis

As mentioned earlier, literature on the effects of PAHs on human health is less than the other environmental chemicals, such as heavy metals, pesticides, arsenic, and phthalates. This is particularly apparent in the relationship of PAHs and digestive conditions.

PAHs have been known as toxic chemicals and could affect renal function. Previously, PAHs were not found to be associated with renal cancer risk in Americans (Karami et al. 2011) but associated with kidney damage in Italians (Lacquaniti et al. 2012). The current findings in the present study were similar to those previous observations showing that PAHs were associated with kidney stones but not failing kidney in American adults. Animal studies using Swiss mouse and rats in vitro also presented that PAHs could damage the kidney (Krajka-Kuźniak and Baer-Dubowska 2003; Roos 2002; Bondy et al. 1995; Bowes and Ramos 1994). In other words, it is likely that PAHs could exacerbate kidney dysfunction but not necessarily lead to fatal events. One of the reasons might be that the study sample was not exposed to the exceeding toxic level. On the other hand, it was observed that people with celiac disease had higher levels of PAHs in the present study. However, no literature has addressed the potential link. Therefore, no comparison could be made and discussed.

### Strengths and limitations

The present study has a few strengths. First, this study was conducted in a large and nationally representative population with mixed ethnicities and socioeconomic status. Second, this is the first time to examine the risk effects of urinary PAHs on adult digestive conditions. However, there are also a few limitations that cannot be ignored. First, there could be still other emerging chemicals from the living environments through different channels/vehicles that we might not yet know and would need future research to further identify and examine. Second, other subtypes of disease of the digestive system were not available in the current limited dataset nor clinical measures for these specific diseases. Third, some disease events, such as celiac disease, were still suffering from small number in the statistical analysis due to the fact that it is not a very common disease. Therefore, some associations could be underestimated. Fourth, causality cannot be established in the present study due to the cross-sectional study design in nature. Future studies with a longitudinal study design to confirm or refute the current findings and, if at all, to understand the persisting risk effects along the life course from those environmental chemicals mentioned above would be suggested.

### Research, practice, and policy implications

In summary, urinary PAHs were positively associated with adult celiac diseases and kidney stones. From the research perspective, longitudinal monitoring from observational studies and experimental research understanding mechanism would be suggested. From the law and human health perspectives, regulation of minimizing PAHs exposure for humans might need to be considered in future health and environmental policies and intervention programs.
